# Comparative life cycle assessment of environmental impacts and economic feasibility of tomato cultivation systems in northern plains of India

**DOI:** 10.1038/s41598-024-57623-9

**Published:** 2024-03-25

**Authors:** Rohit Kumar, Arvind Bhardwaj, Lakhwinder Pal Singh, Gurraj Singh, Anupam Kumar, Kanhu Charan Pattnayak

**Affiliations:** 1 Department of Industrial and Production Engineering, Dr. B. R. Ambedkar National Institute of Technology, Jalandhar, Punjab 144027 India; 2 Centre for Climate Research Singapore, Meteorological Service Singapore, National Environment Agency, Singapore, 537054 Singapore; 3 School of Earth and Environment, University of Leeds, Leeds, LS29JT UK

**Keywords:** Open field tomato, Greenhouse gas emissions, Life cycle assessment, Life cycle costing, Sustainable agriculture, Climate-change ecology, Phenology, Climate-change adaptation, Environmental economics

## Abstract

To meet the growing demand for vegetable production and promote sustainable agriculture, it is imperative to implement effective input management and adopt eco-friendly farming practices. This study aims to compare the environmental impacts of conventional and organic tomato cultivation in the northern plains of India. This study utilizes SimaPro 9.1.1 software for a comprehensive cradle-to-farm gate Life Cycle Assessment (LCA), assessing production stages, identifying key environmental factors, and incorporating ReCiPe Midpoint and Endpoint methods with one-hectare as a functional unit. Findings reveal that conventional cultivation is more affected by fertilizer application and transplanting, while organic cultivation emphasizes transplanting and irrigation. Organic cultivation contributes 904.708 kg CO_2_, while conventional cultivation contributes 1307.917 kg CO_2_ to Global Warming potential. Switching to organic cultivation leads to a significant 35.04% decrease in all impact categories. Using the endpoint method, organic cultivation achieves a notable 27.16% reduction, scoring 58.30 compared to conventional cultivation's 80.04. The LCA analysis of tomato cultivation highlights Fertilizer application as the predominant environmental concern, emphasizing the need for sustainable techniques to minimize waste and mitigate environmental impacts. This study recommends imposing restrictions on fertilizer and pesticide use and formulating effective policies to promote the adoption of sustainable practices.

## Introduction

Agriculture, being a multifaceted and intricate system, gives rise to substantial environmental strains, spanning from the depletion of natural resources to the generation of waste. These burdens predominantly emanate from the widespread adoption of intensive agricultural practices and the application of novel techniques^1^. The agricultural sector plays a crucial role as the first step in the food supply chain, encompassing impact categories such as ecology, geography, soil characteristics, erosion, and freshwater ecosystem. Agriculture production needs massive quantities of capital such as water, fossil fuels, and agrochemicals, whose utilization degrades the ecosystem in various ways^2^. Excessive pesticide use leads to greenhouse gas (GHG) emissions and water pollution^3–5^. In India, freshwater resources are being polluted through drainage and leaching of nitrates from agricultural land and the overuse and misuse of chemical pesticides^6,7^. All of these challenges are closely tied to the fundamental aspect of food safety, prompting extensive societal discussions on how best to address these concerns globally. The United Nations has also included health and food security as a top priority among its Sustainable Development Goals and established various targets to address this issue using existing resources^8^. Additionally, emissions arising from agricultural activities demonstrate high variability due to factors such as local climate, soil quality, agricultural practices, and numerous interconnected elements^9^.

In recent years, there has been a notable increase in global demand for organic crops due to consumer preferences for healthier and more sustainable food choices. A compelling meta-analysis, based on an examination of 343 peer-reviewed publications, highlighted that organic crops tend to have higher concentrations of antioxidant compounds, lower levels of cadmium, and reduced incidence of pesticide residues in their edible parts compared to non-organic crops^10^. However, it is important to note that consumers often lack access to reliable information regarding the true environmental impacts of organic farming, as well as other cropping systems^11^. Consequently, comprehensive studies encompassing various crops are necessary to verify whether the promised benefits of enhanced sustainability and reduced GHG impacts associated with organic farming are genuinely realized or not.

**Figure 1:**
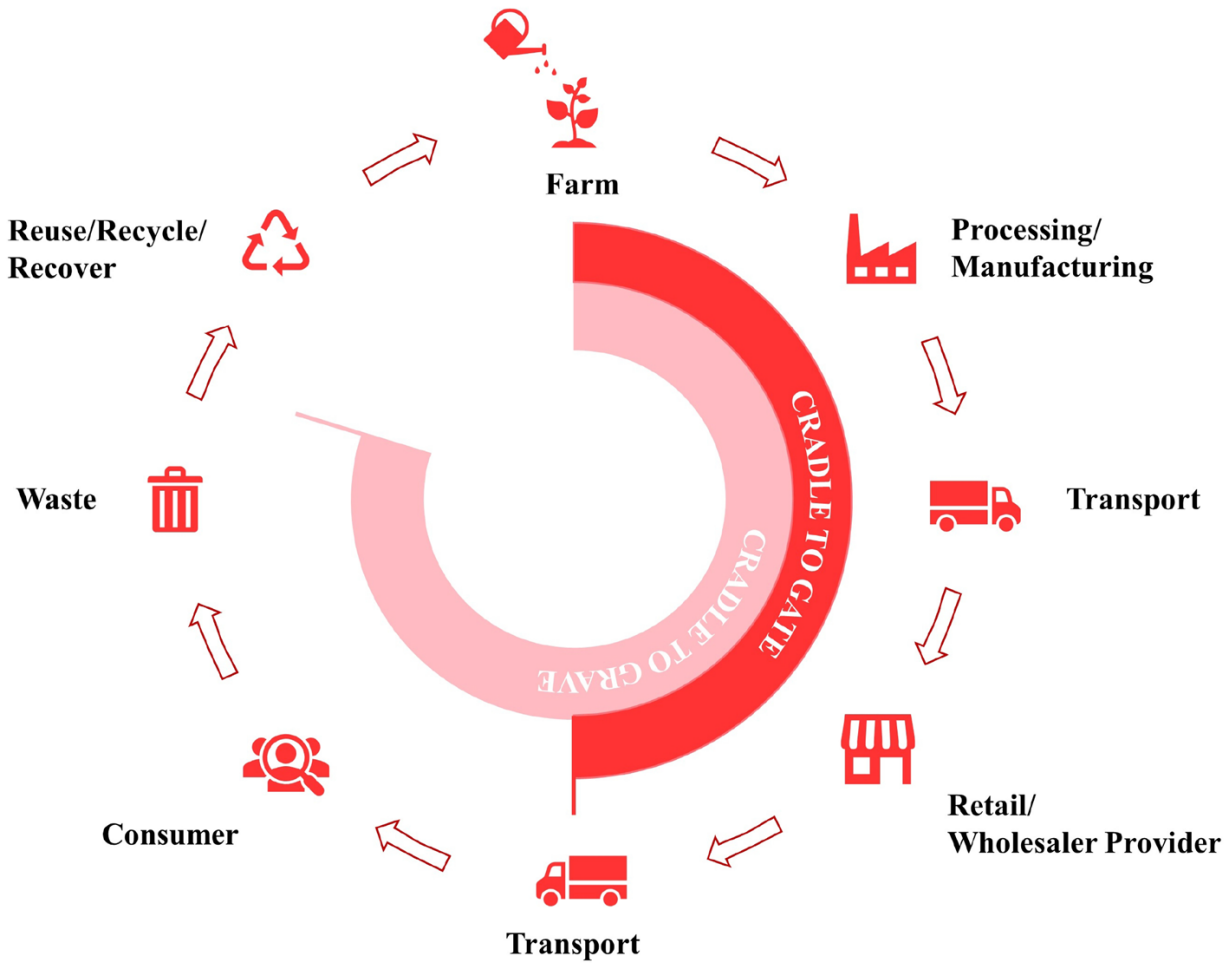
Typical life cycle assessment of a product.

Considering the present scenario, it is essential to satisfy the escalating need for vegetable production while also promoting sustainable agriculture. This necessitates the implementation of an effective approach to manage inputs and adopt eco-friendly farm practices^11–14^. A substantial amount of research has been undertaken and is still in progress to assess farming practices and investigate the overall environmental impact of agriculture, employing diverse methodologies. Among the variety of evaluation methods, LCA is known to be one of the most informative tools for evaluating the environmental impacts of farm products^15^. LCA offers a holistic methodology to analyze the environmental impacts associated with a product across its complete life cycle. Figure 1 showcases the typical stages involved in the life cycle of a certain product. This methodology has established itself as a crucial tool for assessing and contrasting the environmental impacts of diverse agricultural systems. One prominent area of study within LCA revolves around comparing organic and conventional farming practices^16^.

In various industries, including agriculture, experts involved in LCA have increasingly recognized the importance of considering not only environmental factors but also economic and social aspects. One commonly utilized economic approach that complements LCA is Life Cycle Costing (LCC). While the fundamental principles of LCC are still being debated and specific databases for LCC are not yet available, researchers occasionally find themselves adapting their methods within the framework of LCA to maintain consistency in terms of the functional unit and system boundary. Despite this challenge, there is limited existing literature that explores the integration of economic considerations with agricultural production in LCA^12,17,18^.

**Figure 2:**
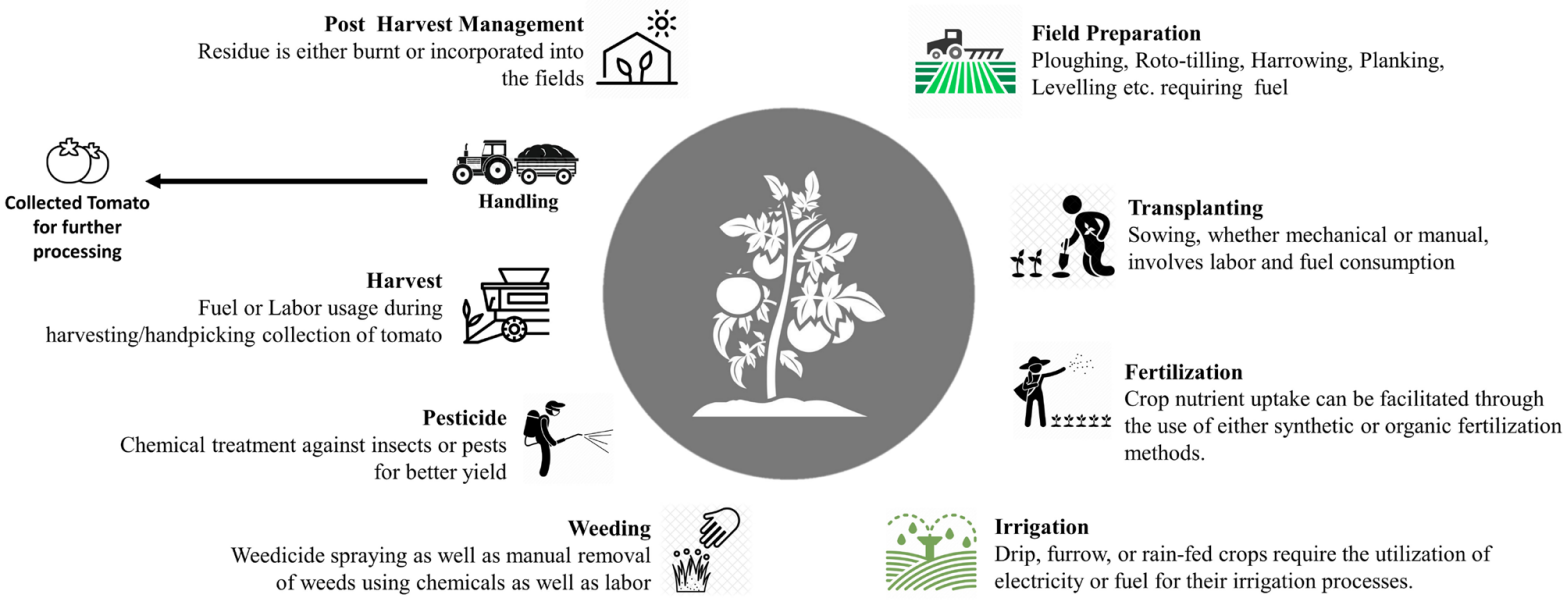
A typical life cycle of a tomato, depicting the sequential stages from seed to tomato.

Tomato (*Solanum* *lycopersicum*) is a popular crop worldwide and belongs to the nightshade family, Solanaceae. Tomatoes are rich in essential nutrients like vitamins A, C, and K, as well as lycopene, an antioxidant associated with various health benefits^19^. Tomatoes thrive in areas with warm temperatures, ideally between 70–85 °F (21–29 °C) during the day and above 50°F (10 °C) at night. They require well-drained soil with good fertility. Sandy loam and loamy soils with a pH range of 6.0–7.0 are suitable for tomato cultivation. Although tomato holds the position of being the second most economically valuable crop and the top processed vegetable globally^20^, it serves a crucial role in the human diet^21^. Figure 2 illustrates the complete life cycle of a tomato crop, including all stages from seed to fruit. The growing demand for vegetable production puts pressure on crops to increase their yields through the use of agrochemicals. However, the extensive use of agrochemicals places a significant burden on the environment, affecting human health, resource utilization, and ecosystem integrity.

Several previous research studies have focused on conducting LCA specifically for the cultivation stage of agricultural production. These studies have yielded noteworthy findings regarding the substantial impact of certain factors. For instance, Del Borghi et al.^22^ and De Marco et al.^23^ have identified the significant contribution of fossil fuels, particularly diesel, to the environmental footprint of cultivation. In their study, Williams et al.^24^ performed an LCA comparing organic and conventional cultivation of crops (wheat, oilseed rape, potatoes, and fresh market tomatoes). Overall, the organic system had 27% lower GHG emissions per unit of product across various crops. However, when focusing on greenhouse-grown tomatoes, the organic system exhibited 30% higher emissions per unit of product, mainly due to lower yields in organic cultivation. A study conducted by He et al.^21^ compared the LCA of greenhouse tomato production in China's organic and traditional systems. The study found that the organic system exhibited a significant 54.87% lower environmental impact compared to the traditional system. This reduction was primarily attributed to the potential decrease in the use of synthetic fertilizers and pesticides. Elnaz et al.^25^ conducted a study comparing the environmental impact of four tomato production scenarios, including greenhouse organic, greenhouse conventional, open field organic, and open field conventional systems. By using LCA, they found that the greenhouse conventional system had the highest yield, while the greenhouse organic system had the lowest environmental damage, suggesting that compost use in the organic open-field scenario had negative environmental effects. Ronga et al. conducted a study comparing the environmental impact of organic and conventional processing tomato production in Southern Italy. They found that the organic system had a higher global warming potential (GWP) when considering 1 ton of tomatoes but lower GWP when considering 1 hectare. The study highlighted the need to improve the environmental efficiency of practices like pesticide use and soil tillage in organic tomato farming and emphasized the importance of reducing the yield gap between organic and conventional systems for sustainability. Despite these considerations, several prior studies have been undertaken to analyze the crop's environmental impacts. These studies have been conducted in various locations, including Spain^26,27^, Canada^28^, Italy^29^, southern and central Europe^30^, and Australia^31^.

Other researchers have conducted comparisons to evaluate the environmental and agronomic performances of various production systems, including greenhouses, shaded structures, and open fields, in different regions around the developed world^26,32–34^. However, there is a noticeable scarcity of studies that investigate the comparison between different cultivation practices, such as organic and conventional methods, especially in developing countries, especially the agricultural giants like India. An imperative study is needed to comprehensively examine the comparison between organic and conventional tomato cultivation systems, encompassing a wide array of environmental impact indicators. Such results may then be further incorporated over a series of other crops.

Moreover, it's worth highlighting that most of the existing research has primarily centered on European countries, where farm sizes tend to be larger^35^, and technological advancements are more prevalent^36^. In addition, prior investigations into tomato production systems have mainly focused on the overall environmental impact of the entire production process, with limited attention given to specific field operations. Consequently, the present study aims to bridge this gap by specifically honing in on the agricultural practices in the fields within both organic and conventional tomato systems, with a particular focus on medium-sized farmers in developing nations. The primary objective is to pinpoint the practices that make the most significant contributions to environmental impacts and explore opportunities for optimization. Moreover, this study incorporates an analysis of LCC to assess the economic aspects of agricultural practices and the overall system. By amalgamating these dimensions, a comprehensive assessment encompassing both environmental and economic factors can be realized.

Based on the above, the objectives of this study are:

- To conduct a comprehensive and simultaneous analysis of the ecological as well as the economic impacts associated with two different tomato cultivation systems (organic and conventional) throughout their growing life cycle.
- To identify specific hotspots within each system that have significant ecological and economic aspects in order to explore potential opportunities for optimizing tomato agricultural practices.

## Materials and methods

### Study area

**Figure 3:**
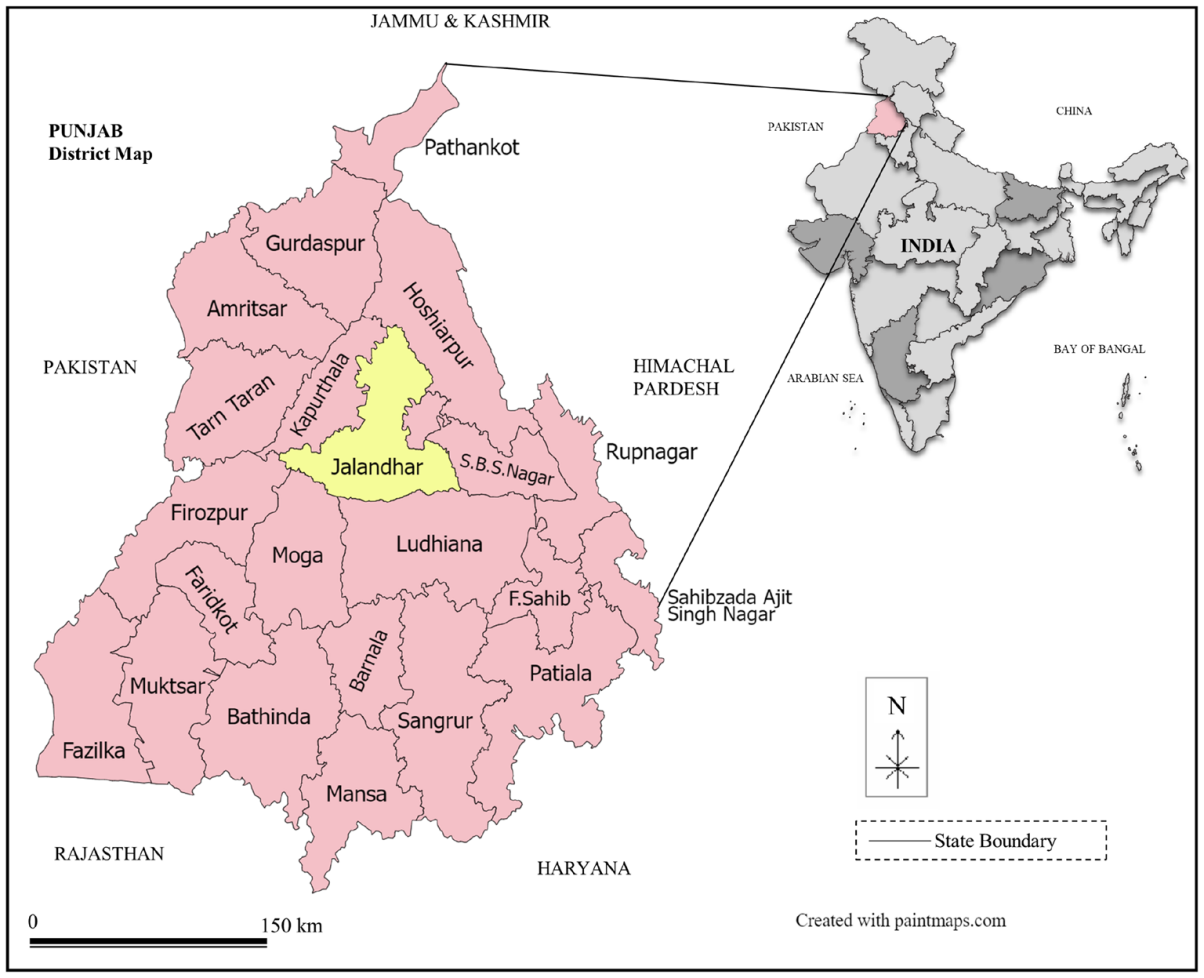
Location of the studied region in the Northern plain of India.

The study conducted its analysis by gathering data from tomato farms located within the Jalandhar district of the Punjab province in India as depicted in Fig. 3. Extensive prior research has consistently highlighted the suitability of this region for tomato cultivation^37,38^. This is attributed to the ideal climate and soil conditions that promote the cultivation of healthy tomatoes during a specific period in the autumn season while also reducing infection rates. During the 2022–2023 crop season survey, a deliberate selection process included a total of forty medium/small-sized tomato farms, with an even distribution of twenty farms practicing conventional cultivation methods and the remaining twenty using organic cultivation methods. The questionnaire was developed to collect foreground data, including information on fertilizers, pesticides, irrigation, seed stems used during planting, transportation distances, and the types of fuels utilized in agricultural machinery during the cultivation phase. The basic questionnaire used in this study has provided as supplementary material (see Supplementary Information). To uphold ethical practices and privacy, we have diligently obtained consent from tomato farmers in the chosen region, enabling us to gather valuable information and conduct essential experiments. Additionally, farms of similar sizes (less than 5 hectares) whose employ identical agricultural practices were chosen for the current study.

### Life cycle assessment

The life cycle assessment has been employed to quantify the environmental burdens of the analyzed systems. The tests, calculations, and analysis of the results adhered to the guidelines set forth by ISO 14040^39^ and ISO 14044^40^ for comprehensive environmental assessments.

#### Goal and scope of the study

This study aims to compare the environmental effects of conventional and organic tomato farming in the northern plains of India while identifying key areas of concern. The primary objective is to determine whether cultivating one hectare of organic tomatoes has a lesser environmental impact compared to conventional methods. Furthermore, the study will analyze the economic aspects of both systems to gain insights into their financial implications. The findings from this study can be utilized to promote eco-friendly practices in tomato cultivation and inform government policies. It is worth mentioning that the cultivation of tomatoes shares similarities with eggplant, melons, and cucumber in terms of processes and mechanization. Therefore, the results of this research may also shed light on the environmental impacts of these related crops.

#### Functional unit

The functional unit describes the primary function fulfilled by a product system, providing a reference to which the input and the output data can be standardized in a mathematical sense^41^. The study employed one hectare of tomato production as a functional unit to examine the potential environmental impacts of tomato production, specifically investigating two crucial cropping systems: conventional and organic. Scientific literature highlights that farmlands not only hold significance for agricultural production but also have a substantial environmental impact at a regional scale, particularly concerning area-based emissions^42^. This underscores the importance of studying the environmental implications of agricultural practices beyond their immediate agricultural productivity.

#### System boundaries

**Figure 4:**
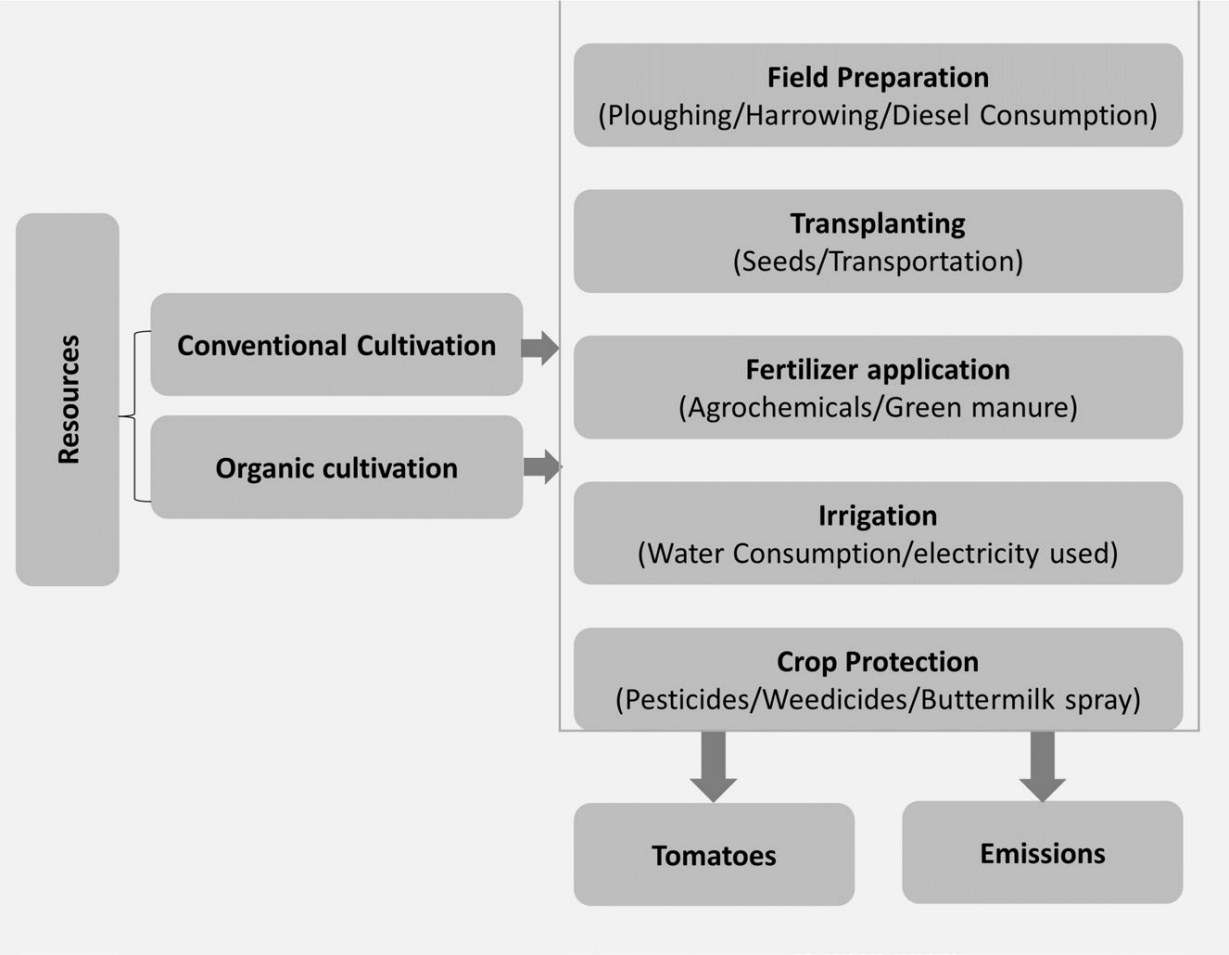
The system boundaries for open-field tomato cultivation encompass the included processes.

The current study adopts a comprehensive approach, known as a cradle-to-farm gate boundary system, which encompasses all activities from land preparation to harvesting. This means that every step involved in the process, as depicted in Fig. 4, has been taken into account. The study thoroughly examined every step involved in tomato production, including both the organic system and the conventional system. Additionally, the study considered all the inputs required for each agricultural operation.

#### Life cycle inventory

The life cycle inventory (LCI) constitutes a crucial component of this study, as it serves as the foundation for subsequent processes. This phase involves the collection of data, identification of interconnections, and quantification of inputs and outputs within the system under evaluation. The LCI provides essential information for further analysis and evaluation throughout the study. Table 1 presents the average utilization of inputs and materials in both cultivation systems.

**Table 1:** Use of production resources per hectare for cultivation of conventional and organic tomato.

Farming practices	Activity	Conventional	Organic	Unit
Field preparation	Transformation from agriculture	1	1	ha
	Transformation to agriculture	1	1	ha
	Ploughing	1	1	times/hectare
	Rototilling	1	1	times/hectare
	Diesel	19.5	19.5	L
Transplanting	Seeds	3	3	kg
	Transportation from market	20	20	km
Fertilizer application	Urea	100	–	kg
	Potassium	115	–	kg
	Potash	100	–	kg
	Organic manure	–	20,000	kg
Crop protection	Mancozeb	4.5	–	kg
	Benzimidazole	0.009	–	kg
	Chlorpyrifos	2.5	–	kg
	Sour buttermilk	–	12.5	L
	Water	–	1	m^3^
	Fuel consumption in power sprayer	2.5	0.9	L
Irrigation	Water	440.2	560.2	m^3^
	Electricity	65.15	80.15	kWh
Output	Yield	28	15	tonnes/hectare

For tomato plantation, the field preparation involves thoroughly pulverizing and leveling the soil. To achieve a fine tilth, the land is plowed multiple times (around 4–5 times), followed by planking to ensure soil leveling. In organic cultivation, an additional step is taken during the last plowing, where well-decomposed cow dung is applied to the soil. As part of the LCA, inputs such as diesel used in agricultural machinery during field preparation have been documented. In the case of tomato cultivation in northern states for the autumn crop, sowing typically takes place in July–August, followed by transplantation in August–September. It was observed that an average seed rate of 250 g was used for preparing seedlings for sowing on one hectare of land. During the fertilizer application, the following quantities were applied per hectare: Urea at 100 kg/ha, Single Super Phosphate at 100 kg/ha, and MOP (Muriate of Potash) at 115 kg/ha. In contrast, in the organic fields, an average of 20 tons per hectare of solid cattle manure was utilized. The whole manure was applied within a week of transplanting the tomato seedlings, allowing time for soil organic matter enrichment. This organic fertilizer was transported from nearby dairy farms using a tractor and trailer. The transfer of manure from the source to each tomato field had a mean distance of 25 km. Similarly, in conventional cultivation, the mean distance for the transportation of fertilizers and pesticides from the point of procurement to the farm was recorded as 20 km. All the data collected for these distances were based on the mean values obtained from the farmers participating in the study.

In conventional cultivation, crop protection was primarily achieved through the use of synthetic herbicides and fungicides. However, in organic cultivation, sour buttermilk was employed as a pesticide. The study recorded all the materials and inputs used for the life cycle analysis, including the quantity of pesticides, fuel consumption for agricultural operations, and water usage during spraying. Regarding irrigation, the water requirements for tomatoes differ based on the cultivation method. Conventional cultivation typically involves three irrigation cycles, while organic cultivation necessitates more irrigation due to the longer duration of the crop. All farmers used electricity to operate their water pumps, and the study took into account the average electricity consumption across the selected farms. The average yield of both cultivation systems was documented, revealing a disparity of approximately 37 percent (24 tons for conventional and 15 tons for organic). The reduced yields in organic cultivation can be attributed to the lower utilization of external inputs and the increased presence of pests and weeds, as indicated by Ronga et al.^41^. The necessary data that was unavailable was obtained from the widely recognized Ecoinvent version 3.7 database, which is accessible through SimaPro software. For the sake of simplicity, this study did not incorporate the environmental impacts of producing farm-used capital goods, such as farm facilities and equipment depreciation. This decision was based on previous research^43,44^, which indicated that the long lifespan of such goods does not significantly impact a single production. The direct emissions resulting from fertilizer usage have been estimated based on data from previous studies, as indicated in Table 2. However, it should be noted that due to limited data availability regarding the proportion of eroded soil, the release of phosphates into surface waters through erosion was not included in the calculations. Moreover, consistent with the findings of Blanco et al.^26^ suggested that the discharge of phosphates into surface waters may not result in a substantial environmental impact on cultivation of tomatoes.

**Table 2:** Output Life cycle inventory for selected boundary system of per hectare of tomato cultivation.

Output	Quantity
NH_3_ emissions	3% total N applied^45^
N_2_O emissions	1.25% total N applied^45^
NO_x_ emissions	10% total N_2_O emissions^46^

#### Life cycle impact assessment

To evaluate the environmental connections between inputs and outputs in the LCA research and estimate their impact, the study utilized SimaPro version 8.1.0 software. This software, in conjunction with the Ecoinvent database, facilitated the analysis and prediction of the environmental implications based on the collected data. The assessment of emissions was conducted using the ReCiPe methodology, which includes midpoint and endpoint indicators^47^. The endpoint indicators assess the impact on the environment across three higher aggregation levels: human health, biodiversity, and ecosystem, and resource scarcity^48^. By utilizing these two distinct perspectives, the results were examined comprehensively to provide a more complete understanding of the environmental impacts associated with conventional and organic tomato cultivation. This approach allows for a robust analysis of the potential effects across various environmental indicators, providing a more comprehensive and reliable assessment of the cultivation systems.

## Result and discussion

The primary objective of this study was to determine the key factors that could significantly impact the LCA results of both systems of cultivation i.e., conventional and organic tomato cultivation. The aim was to establish connections between various agricultural practices that have a lesser detrimental effect, in order to better understand the overall environmental impacts of different cultivation systems. It is vital to examine the specific impact of each agricultural activity on the overall life cycle impacts of both cultivation systems.

### Interpretation of midpoint characterization results w.r.t agricultural practices

**Figure 5:**
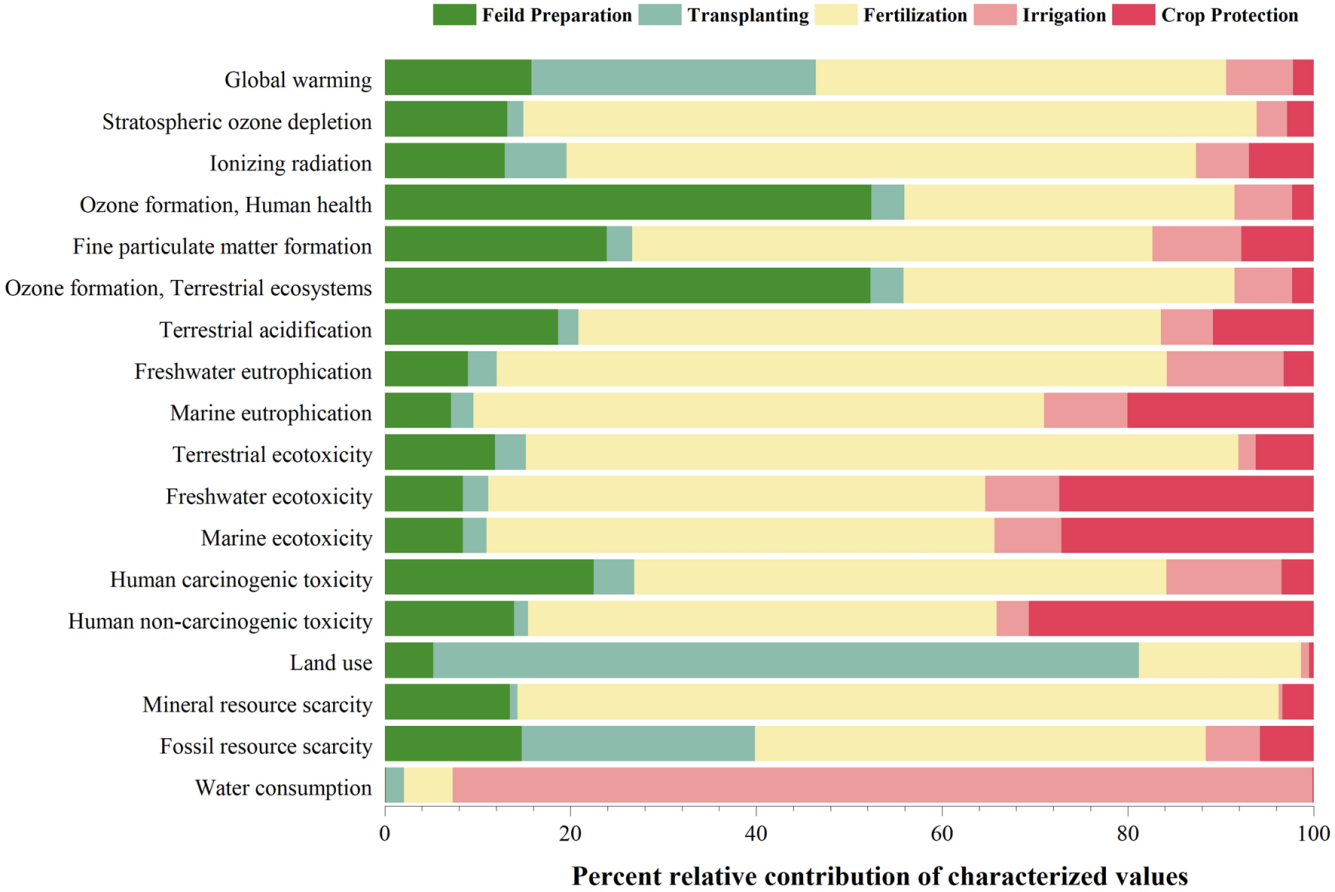
Characterization value of conventional tomato production based on the ReCiPe Midpoint method.

**Figure 6:**
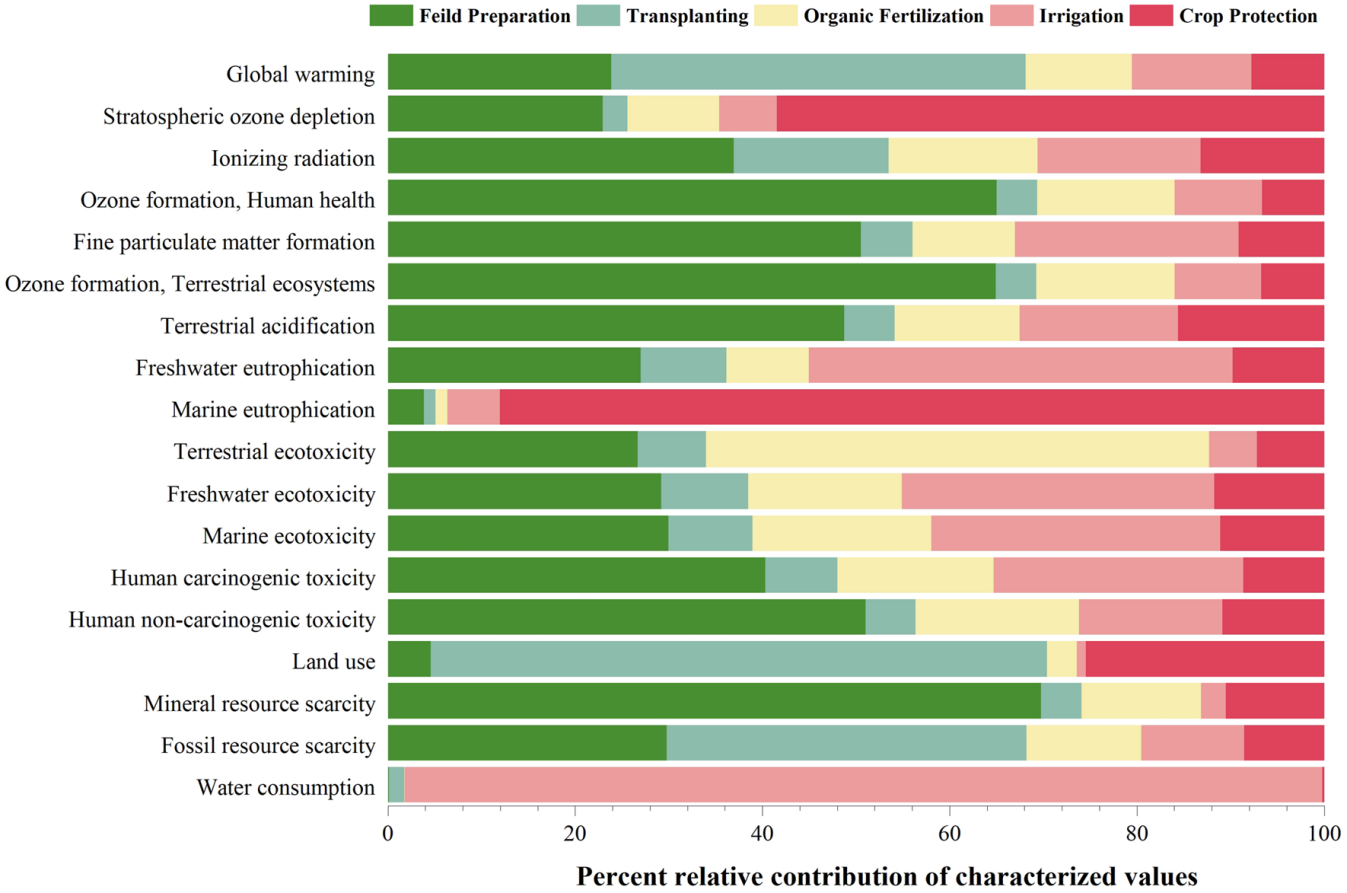
Characterization value of organic tomato production based on the ReCiPe Midpoint method.

In Figs. 5 and 6, the variation in the results and environmental effects of tomato cultivation is illustrated through the ReCiPe Midpoint characterization. The graphs provide a distinct view of the outcomes for each cultivation system. The graphical representation demonstrates that specific farming practices, including Fertilizer application, irrigation, transplanting, and field preparation, have notable environmental impacts. Firstly, it is evident that the Fertilizer application and transplanting stages exhibit a greater influence compared to other phases. This observation aligns with existing historical data that emphasizes the significant contribution of the Fertilizer application phase in similar agricultural processes^29,49–51^. In conventional tomato cultivation, the Fertilizer application phase stands out as the key contributor to the overall impact, largely attributed to the production and transportation of fertilizers, which heavily depend on fossil fuels^52^. This aligns with the findings of previous studies, indicating that conventional farming practices prioritize maximizing yield in economically viable ways, with some consideration given to environmental factors^53^. The environmental impact of irrigation is primarily negative, mainly because of its excessive freshwater usage for crop hydration and its reliance on coal-based electricity^54^, which intensifies resource consumption^55^. However, in comparison to other practices, irrigation’s contribution is relatively limited since it relies less on inputs from nature and technology. Nevertheless, the remaining cultivation practices also exert a substantial impact due to the utilization of resources and inputs.

In the realm of organic tomato cultivation, it has been determined that the field preparation and transplantation stages exert a more substantial influence compared to other practices such as organic Fertilizer application, crop management, and irrigation. Furthermore, an intriguing observation emerged, indicating that irrigation practices in organic cultivation possess an approximately 18.85% greater impact compared to conventional cultivation. This discrepancy primarily arises due to the prolonged lifespan of organic crops, which necessitates higher water consumption when contrasted with conventional crops. Consequently, escalated water usage contributes to the heightened environmental repercussions associated with irrigation in organic cultivation.

### Interpretation of midpoint characterization results w.r.t impact categories

In terms of GWP, the organic cultivation method demonstrated a significantly lower impact, with a reduction of approximately 40% compared to the conventional system. The conventional system exhibited a GWP of 1307.917 kg CO_2_ eq per hectare, while the organic system recorded 904.708 kg CO_2_ eq per hectare. When considering the contribution of individual stages in the management process, the application of fertilizer had the highest impact in the conventional system, accounting for approximately 44% of the total. Other stages such as transplantation, field preparation, irrigation, and crop protection contributed 30.63%, 15.80%, 7.17%, and 2.22% respectively. It is evident that higher nitrogen and phosphorus usage contributes to increased GHG emissions. The earlier research also provides substantial evidence supporting the relationship between greenhouse gas emissions and the amount of synthetic nutrients used in crop production^4,56,57^. Naseer et al.^58^ also investigated Year-round production in southwestern Norway demonstrated a noteworthy decrease in environmental impact across multiple categories, with greenhouse heating identified as a major contributor. However, in the context of organic farming, field preparations were found to have the highest impact, accounting for 44.28% of the total. This was followed by field emission activities at 23.84%, irrigation at 12.76%, organic fertilizer at 11.36%, and crop protection at 7% the stage with the lowest impact within the GWP category. The significant reduction in fertilizer impact, compared to the conventional system, aligns with the findings of Ronga et al.^41^. Their research emphasized the lower GWP contribution of organic cultivation, with a substantial 40% lower impact on average per 1 hectare of production, compared to conventional (3154.03 kg CO_2_-eq per ha in organic vs. 5290.74 kg CO_2_-eq per ha in conventional of GHG emissions). Similarly, Solimene et al.^59^ conducted a LCA on fresh tomatoes, comparing three business models at the farm gate. Results revealed a significant contrast, with a higher GWP of 5542 kg CO_2_-eq per ha in standard production (with increased agrochemical use) compared to 1600 kg CO_2_-eq/ha in precision farming. Fertilizer use emerged as the major contributor to climate-changing gases, highlighting precision farming’s noteworthy reduction in CO_2_ emissions and providing insights for sustainable large-scale tomato production in Italy. Discrepancies in results were attributed to greater resource consumption as well as unique geographical features. This further supports the environmental benefits associated with alternative cultivation practices. Moreover, as per Longo et al.^51^ a significant amount of the influence on the natural ecosystem is attributable to the use of fertilizers, insecticides, and fuel by agricultural machinery.

The comprehensive analysis of impact indicators beyond GHG emissions, as presented in Table 3, sheds light on the intricate environmental implications associated with different agricultural practices. Beyond the noteworthy reduction in GHG emissions observed in organic cultivation, the focus on additional impact categories such as terrestrial ecotoxicity, human toxicity, and fossil resource scarcity provides a more nuanced understanding of the overall sustainability of farming systems. Notably, the Fertilizer application phase stands out as the major contributor, responsible for over 40 to 60 percent of the impact in each category. This significant influence can be attributed to the production, transportation, and application of fertilizers during this particular phase. The extensive use of synthetic fertilizers significantly increased the ecotoxicity of the ecosystem^60,61^ which led to an increase above impact categories.

**Table 3:** Midpoint characterization values of both cultivation systems.

Impact category	Unit	Field preparation		Transplanting		Fertilizer application		Irrigation		Crop protection		Total		%Change
		Conventional	Organic	Conventional	Organic	Conventional	Organic	Conventional	Organic	Conventional	Organic	Conventional	Organic	
Global warming	kg CO_2_ eq	206.715	215.754	400.646	400.646	577.576	102.787	93.870	115.482	29.110	70.039	1307.917	904.708	30.83
Stratospheric ozone depletion	kg CFC11 eq	1.08E−04	1.23E−04	1.41E−05	1.41E−05	6.47E−04	5.25E−05	2.68E−05	3.30E−05	2.34E−05	3.13E−04	8.19E−04	5.36E−04	34.57
Ionizing radiation	kBq Co-60 eq	4.576	5.276	2.362	2.362	24.063	2.269	2.022	2.488	2.470	1.885	35.493	14.281	59.76
Ozone formation, Human health	kg NOx eq	1.850	1.882	0.125	0.125	1.255	0.425	0.219	0.270	0.081	0.192	3.530	2.894	18.01
Fine particulate matter formation	kg PM2.5 eq	0.599	0.625	0.068	0.068	1.405	0.135	0.240	0.295	0.195	0.113	2.507	1.237	50.66
Ozone formation, Terrestrial ecosystems	kg NOx eq	1.880	1.914	0.128	0.128	1.282	0.435	0.221	0.272	0.084	0.198	3.595	2.947	18.01
Terrestrial acidification	kg SO_2_ eq	1.041	1.117	0.123	0.123	3.502	0.306	0.315	0.387	0.603	0.358	5.583	2.290	58.98
Freshwater eutrophication	kg P eq	0.035	0.036	0.012	0.012	0.286	0.012	0.050	0.061	0.013	0.013	0.397	0.135	65.93
Marine eutrophication	kg N eq	0.002	0.003	0.001	0.001	0.021	0.001	0.003	0.004	0.007	0.058	0.034	0.066	− 96.81
Terrestrial ecotoxicity	kg 1,4-DCB	538.377	550.440	151.325	151.325	3483.867	1109.473	85.674	105.399	282.285	147.848	4541.529	2064.485	54.54
Freshwater ecotoxicity	kg 1,4-DCB	3.416	3.499	1.112	1.112	21.800	1.968	3.252	4.000	11.140	1.404	40.720	11.983	70.57
Marine ecotoxicity	kg 1,4-DCB	4.928	5.074	1.520	1.520	32.121	3.239	4.247	5.225	15.943	1.881	58.759	16.939	71.17
Human carcinogenic toxicity	kg 1,4-DCB	7.556	7.746	1.480	1.480	19.256	3.205	4.164	5.123	1.153	1.661	33.609	19.215	42.83
Human non-carcinogenic toxicity	kg 1,4-DCB	202.995	205.560	21.433	21.433	735.349	70.428	50.168	61.719	446.391	43.748	1456.337	402.888	72.34
Land use	m2a crop eq	6.103	6.211	89.072	89.072	20.386	4.344	1.020	1.255	0.594	34.461	117.174	135.343	− 15.51
Mineral resource scarcity	kg Cu eq	1.515	1.530	0.094	0.094	9.222	0.281	0.047	0.058	0.377	0.230	11.255	2.193	80.51
Fossil resource scarcity	kg oil eq	64.192	84.755	109.361	109.361	211.540	34.931	25.360	31.199	25.102	24.321	435.554	284.566	34.67
Water consumption	m^3^	0.594	0.694	9.321	9.321	24.761	0.320	440.510	560.581	0.584	1.161	475.770	572.077	− 20.24

Conversely, in organic cultivation, the transplanting of tomato plants assumes a significant role in terms of impact. This can be attributed to the transportation and utilization of resources and materials preceding the transplanting phase. Notably, the shift to organic cultivation demonstrates an average reduction of 35 percent across all categories, except for Marine Eutrophication and Land use. The amplified contribution to marine eutrophication is primarily attributed to the elevated water consumption and electricity usage involved in pumping water from ground level^62,63^. The current study’s results are consistent with He et al.^21^ findings, where they observed a significant 54.87% lower environmental impact in the organic system compared to the traditional system. This decrease was primarily associated with the potential reduction in synthetic fertilizer and pesticide use. Furthermore, advocating for organic nutrient management is considered a means to reduce adverse environmental impacts while simultaneously improving soil quality, biodiversity, human health, and ecosystem quality^59,64^.

### Interpretation of endpoint results

**Figure 7:**
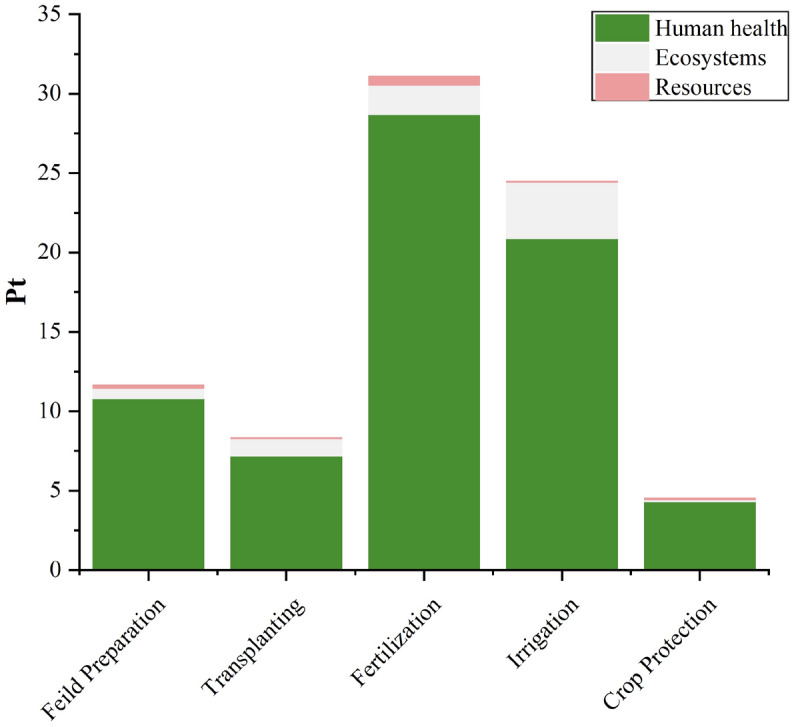
Endpoint score results with respect to conventional cultivation of tomato.

**Figure 8:**
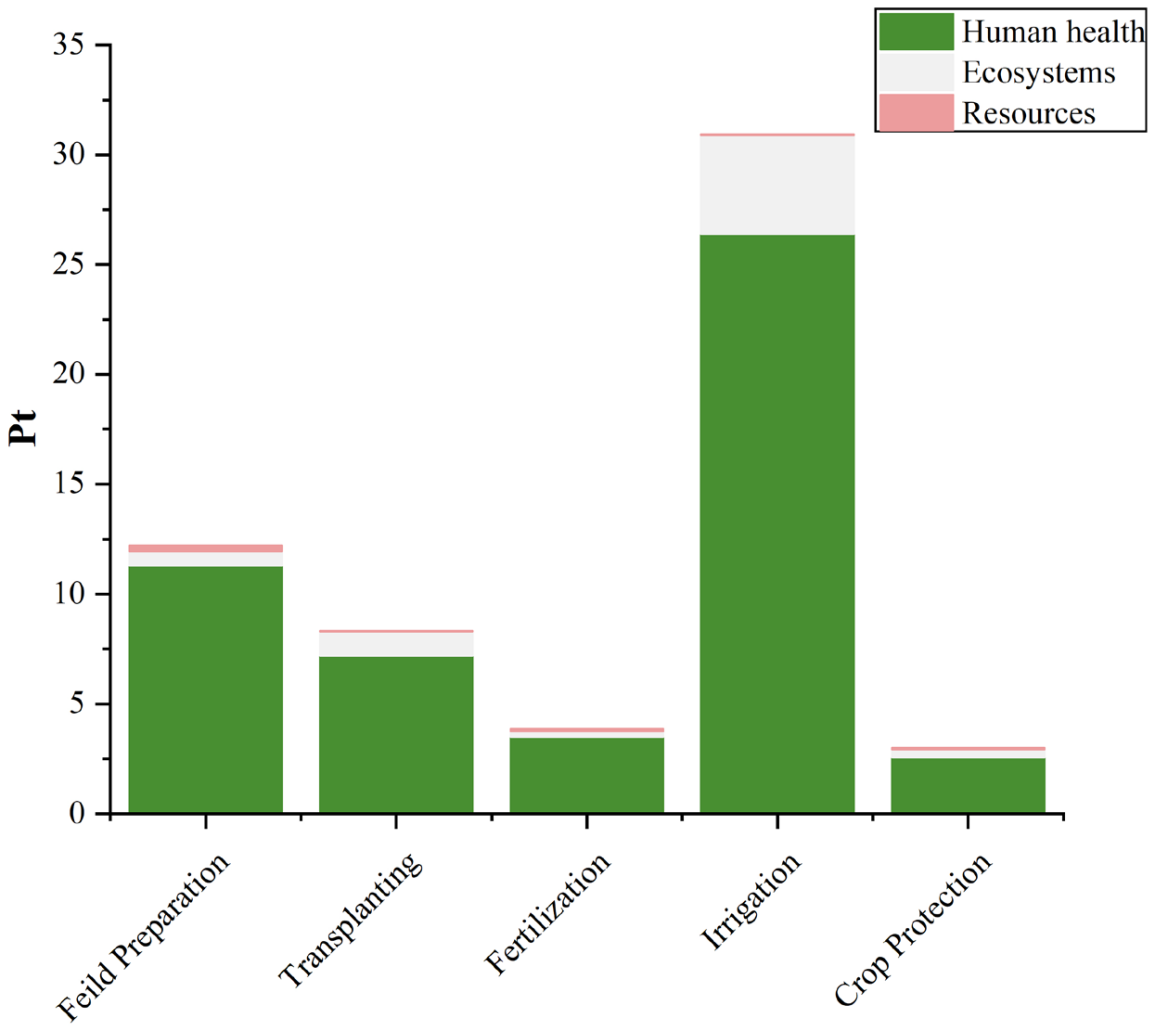
Endpoint score results with respect to organic cultivation of tomato.

Figures 7 and 8 illustrate the variation in the results of tomato cultivation based on the ReCiPe Endpoint score. These figures visually represent the diverse outcomes and effects observed in different environmental categories as a result of tomato cultivation. The final results obtained from aggregating the individual impacts of various agricultural practices in conventional tomato cultivation are presented in Table 4. The combined score represents a single-point assessment, with a total impact of 80.04 points. Among the different categories, Human health has the highest score, accounting for 71.94 points and thus holding a dominant position. Fertilizer application and irrigation practices stand out prominently, with scores of 31.08 points and 24.47 points, respectively. Furthermore, within the score of 31.08 points for Fertilizer application, a significant contribution of 92.38% is attributed to the Human health category. Similarly, within the 24.47 points scored for irrigation, 85.43% of the contribution comes from indicators related to Human health.

**Table 4:** Pointwise score of conventional and organic tomato cultivation.

Damage category	Unit	%age Change	Field Preparation		Transplanting		Fertilizer application		Irrigation		Crop Protection		Total	
			Conventional	Organic	Conventional	Organic	Conventional	Organic	Conventional	Organic	Conventional	Organic	Conventional	Organic
Human health	Pt	29.25	10.82	11.30	7.19	7.19	28.71	3.50	20.90	26.40	4.31	2.58	71.94	50.90
Ecosystems	Pt	5.18	0.63	0.66	1.11	1.11	1.83	0.26	3.55	4.50	0.14	0.35	7.27	6.89
Resources	Pt	43.38	0.18	0.25	0.02	0.02	0.54	0.11	0.02	0.02	0.07	0.07	0.83	0.47
Total	Pt	27.16	11.64	12.20	8.32	8.32	31.08	3.87	24.47	30.90	4.53	3.00	80.04	58.30

In contrast, organic cultivation yields a total score of 58.30 points, which is approximately 27.16% lower than conventional cultivation. Notably, the irrigation phase exhibits the highest point contribution, amounting to 30.90 points, marking a 26.28% increase compared to the conventional phase. The scores for field preparation and transplanting remain unchanged, while there is a significant 87.54% reduction in the total score for the Fertilizer application phase (31.08 points for conventional and 3.87 points for organic). Additionally, the score for the crop protection phase experiences a one-third reduction (4.53 points for conventional and 3.0 points for organic) as a result of the shift from synthetic pesticide spray in conventional cultivation to organic spray in organic cultivation. These findings indicate that organic cultivation substantially diminishes the significance of the Fertilizer application phase, while irrigation becomes a prominent contributing factor due to higher water consumption and the utilization of coal-based electricity.

### Life cycle costing

The analytical method of life cycle costing (LCC) was employed to examine the expenses linked to tomato production throughout its entire cultivation period^18^. The LCC approach was utilized to calculate the costs involved in producing tomatoes, which encompassed various stages for both the selected cultivation systems for tomatoes. Costs were estimated based on market prices of energy sources (diesel and electricity) and materials (chemical fertilizers, herbicides, and insecticides). These prices were obtained from field surveys, reliable government websites, and published literature, focusing on the Indian market. The net economic profit was determined in terms of Indian Rupees per hectare or per unit of output by subtracting the yield price from the total production costs. Moreover, when calculating the costs of agricultural practices and revenues, the following factors were taken into consideration:

- The minimum rate of wage^65^ has been taken for the labor work of removal of weedicide manually. They generally labor more than 8 h per day and are paid between 200 and 400 INR per day, depending on local living conditions.
- The cost of diesel per liter, taken as an average of the selected crop season prices was 89.57 INR in both cultivation systems
- In both systems, the cost of electricity for crop irrigation has been considered zero due to the government of India's policy of providing free electricity to the agriculture sector^66^.
- The fixed capital or indirect costs such as land improvements, indirect labor, and depreciation on machinery and equipment. Interest, rent. farm buildings and work animals have not been considered in this study as the lifespan of such commodities is too long to have a significant impact on single-crop production as stated in earlier studies^46,66^.
- The farm gate price for conventional tomatoes has been set at Rs. 5 per kilogram. However, the price of organic tomatoes can vary depending on factors such as quality, shelf life, and proximity to the sales point. In general, organically produced tomatoes tend to have a higher price, ranging from approximately Rs. 6 to 10 per kilogram. The higher price of organic tomatoes is attributed to their premium market value and the perception of their superior nutritional quality. Since the selling price of organic tomatoes is uncertain, an estimation has been made based on the Benefit to Cost ratio.

Using current market prices of materials and energy sold in the selected regions, the production cost of tomatoes was estimated to be 31,914.93 INR for conventional and 26,086.918 INR for organic cultivation systems (Table 5). The key variable related to the production cost of tomatoes (in the case of conventional) consist of the cost of seedling (27.09%), the combined cost of all pesticides/weedicides used (20.61%), combined the cost of all fertilizers used (18.09%) and cost of Labor expenses (15.04%). In the case of organic cultivation, the cost of transplanting (33.14%) and the cost of labor expenses for the manual removal of weeding (28.75%) lend the major portion of the total cost with a share of 33.14% and 28.75%. It can be seen that the production cost per hectare of organic is lower than that of conventional cultivation (18.26%). For further understanding, the Benefit–Cost Ratio (BCR) is calculated by dividing the Net returns of a selected cultivation system by the total cost of production.

**Table 5:** Costing of all the inputs with respect to both cultivation systems.

Economic consideration		Cost (INR)	
		Conventional cultivation	Organic cultivation
Field preparation	Diesel burning in agriculture machinery	1747.59 (5.48)	1747.59 (6.70)
Transplanting	Seedling	8645 (27.09)	8645 (33.14)
	Transportation of seed from the market	179.24 (0.56)	179.24 (0.69)
	Labor expenses	4800 (15.04)	4800 (18.40)
Fertilizer application	Phosphate fertilizer, as P_2_O_5_	3000 (9.40)	0
	Potassium fertilizer, as K_2_O	2185 (6.85)	0
	Urea, as N	600 (1.88)	0
	Transportation of fertilizers/manure from market/farm	179.24 (0.56)	215.088 (0.82)
Irrigation	Electricity	0	0
Crop protection	Mancozeb	1800 (5.64)	0
	Chlorphriphos + cypermethrin	900 (2.82)	0
	Emamectin benzoate	1110 (3.48)	0
	Diesel burning in sprayer	268.86 (0.84)	0
	Labour expenses for crop protection	2500 (7.83)	7500 (28.75)
Harvesting	Hand Picking/Transportation charges up to the Selling point	4000 (12.53)	3000 (11.50)
Total cost			
INR/hectare		31,914.93	26,086.918
Net sales of crop			
Tomato production (ton per hectare)		24	15
INR/ton		5000	To be estimated

Figure in parentheses indicates percentage to total production cost.

From the economic point of view, it is visible that in order to match the BCR ratio of 2.96 for conventional production at 5 rupees per kg, the cost of organic tomatoes needs to be fixed at 6.5 INR per kg which is 30 percent higher. When focusing on the marketing scenario, the trend of buyers does not seem highly favorable to spend more than 25 to 50 percent excess to the conventional one^46,67,68^. To capitalize on the market potential for organic tomatoes, it is recommended to find a pricing strategy that balances the higher cost of organic production with consumer affordability. Keeping the price difference within the acceptable range of 15 to 30 percent can help attract customers who value organic products and are willing to pay a modest premium for them^68^. Additionally, it is important to consider the marketing strategies based on the size of the farm. Small-scale farms, with limited quantities available, can focus on localized marketing efforts such as word-of-mouth within the local community. However, larger farms with higher production volumes should explore additional marketing channels beyond roadside vendors and word-of-mouth. Allocating a marketing budget for activities like advertisements and expanding sales to other cities can enhance market reach and increase the potential customer base for organic tomatoes. This increases the potential for reaching a wider customer base and commanding better prices, resulting in a higher BCR ratio.

In conclusion, to promote organic tomato production and make it economically viable, it is important to consider pricing strategies that maintain a reasonable price differential compared to conventional tomatoes. Additionally, supporting small-scale farmers with localized marketing efforts and providing larger-scale farmers with resources for wider market reach can improve the overall BCR ratio. Government intervention through registration, subsidiaries, and support programs can play a crucial role in supporting and incentivizing organic farming practices.

## Conclusions

In conclusion, the comparative analysis conducted in this study focused on assessing conventional and organic tomato cultivation systems in Northern India, specifically evaluating their impact on ReCiPe Midpoint and Endpoint impact categories. The key findings reveal that in conventional cultivation, fertilizer application significantly influences the environmental impact, primarily due to reliance on fossil fuels in production and transportation. Additionally, transplanting processes contribute due to diesel fuel consumption. Organic cultivation, however, demonstrates a notable reduction in the impact of fertilizer application across all midpoint impact categories, leading to improvements in various environmental indicators. Switching to organic cultivation results in substantial reductions in mineral resource scarcity, human non-carcinogenic toxicity, marine ecotoxicity, freshwater ecotoxicity, terrestrial ecotoxicity, and global warming potential. The endpoint analysis further highlights a 27.16% reduction in total scores for organic cultivation compared to conventional methods, emphasizing the positive environmental outcomes associated with organic farming practices. Notably, human health remains a dominant concern in both cultivation methods.

The study suggests that minimizing fertilizer usage and incorporating renewable energy are critical for mitigating environmental impacts. The examination of financial viability through a LCC analysis indicates a 20% increase in production costs for conventional cultivation, primarily attributed to expensive fertilizers and pesticides. To improve financial viability, strategies such as integrated pest management, organic fertilizers, and crop rotation are recommended to reduce input costs while maintaining productivity.

The overall LCA underscores the environmental hotspots associated with various agricultural practices, emphasizing the urgency of adopting more sustainable techniques in tomato cultivation. The study advocates for restricting the use of fertilizers and pesticides, implementing thresholds to prevent excessive application, and calls for effective government policies to facilitate affordable access to solar energy equipment for farmers. These measures can enhance the financial viability of alternative practices and contribute to the overall sustainability of the tomato production cycle, aligning with global environmental goals.

### Supplementary Information

Supplementary Information.

## Data Availability

The data that support the findings of this study are available from the corresponding author upon reasonable request.
