# Supplementary material for: Comparative life cycle assessment of environmental impacts and economic feasibility of tomato cultivation systems in northern plains of India
Source: Sci Rep. 2024 Mar 25;14:7084. doi: 10.1038/s41598-024-57623-9 (PMC10963740; doi:10.1038/s41598-024-57623-9)
Supplement: Supplementary file 1 — Supplementary Information. [file 41598_2024_57623_MOESM1_ESM.docx]

**Comparative Life Cycle Assessment of Environmental Impacts and Economic Feasibility of Tomato Cultivation systems in Northern Plains of India**

Rohit Kumar^1^, Arvind Bhardwaj^1^, Lakhwinder Pal Singh^1^, Gurraj Singh^1^, Anupam Kumar^2^ and Kanhu Charan Pattnayak^2,3,*^

Supplementary Information

LCA Questionnaire

**District Code Respondent No.**

**F1.** District Name______________________

**F3.** Parliamentary Constituency (P.C.) Name: ____________________________________

**F4.** Assembly Constituency (A.C.) Name: ______________________________________

**F5.** Village Name: ______________________________________________

**F6.** Name of the Respondent: ________________________________________________

**F7.** Address of the respondent ***(Give landmark)***: _________________________________

____________________________________________________________________

**F8.** Date of interview ***(dd/mm/yyyy)***: ____________________

**F9.** Time of starting the interview: ___________________

**F10.** Name of the Investigator: ____________________________________

**INVESTIGATOR’S INTRODUCTION AND STATEMENT OF INFORMED CONSENT**

My Name is ______________________________ and I have come from the Department of Industrial & Production Engineering, Dr B R Ambedkar National Institute of Technology, Jalandhar (an Institute of National importance By Govt. of India). We are conducting a farmer survey to assess the Life cycle impact assessment of One-hectare tomatoes produced with different cultivation systems (i.e. conventional and organic cultivation). For this exercise we will be interviewing thousands of farmers across the country. The findings of this survey will be used for writing articles in various newspapers and other journals. This survey is an independent study and is not linked to any political party or government agency. Whatever information you provide will be kept strictly confidential. Participation in this survey is voluntary and it is entierly up to you to answer or not answer any question that I ask. We hope that you will take part in this survey since your participation is important. It usually takes 40 to 50 minutes to complete this interview. Please spare some time for the interview and help me in successfully completing the survey.

# Α. GENERAL CHARACTERISTICS

1. Please specify the cultivated area of tomatoes in your farm (in hectares or m^2^)?
2. Number of cultivated tomatoes per hectare (if calculated)?
3. Mean-life of cultivated tomatoes?
4. Is there any co-cultivation of the tomatoes with other cultivation species? If yes, please specify your answer
5. What is the approximate distance between farm and the garage/storage building of your machinery?
6. What is the approximate distance between your farmyard and the storage building of the final product?
7. What is the seed rate used for preparing seedlings for sowing on one hectare of land?
8. What is the average yield of tomatoes per hectare in conventional and organic cultivation?

# Β. AGRICULTURAL PRACTICES

1. Which is the supplier of the chemical fertilizers applied (e.g. Agricultural Association, Agronomist, Shop, Other farmer, etc.)
2. Which is the supplier of the organic fertilizers applied (eg. Agricultural Association, Agronomist, Shop, Other farmer, etc.)
3. What is the distance between your farmyard and the point of procurement of fertilizers (in km).
4. In organic cultivation, what is the quantity of solid cattle manure used per hectare, and how is it transported to the fields?
5. What is the distance between your farmyard and the point of procurement of cattle manure (in km).
6. How many times is the land plowed and leveled during field preparation for tomato cultivation?
7. Types of Agrochemicals used in your tomato field

| N/P/K content per ha | | | |
| --- | --- | --- | --- |
|  | compost | manure | other |
| Ν (Nitrogen) in kg |  |  |  |
| Ρ (Phosphorus) in kg |  |  |  |
| Κ (Potassium) in kg |  |  |  |
| *Total content per*  *ha (in kg)* |  |  |  |

|  | Total content per ha/year (in tonnes  or liters) | Type/Name | Supplier |
| --- | --- | --- | --- |
| Type of fungicide |  |  |  |
| Type of insecticide |  |  |  |
| Type of herbicide |  |  |  |
| Type of bactericide |  |  |  |

# C. IRRIGATION PRACTICES

1. Please specify the type and quantity of the water for irrigation applied in your field.

| ***Irrigation source*** | ***Quantity per ha***  ***(in m^3^)*** |
| --- | --- |
|  Domestic  (Water, surface) |  |
|  Private  (Groundwater) |  |
|  Cistern/Water  Reservoir |  |
|  Other, please  specify |  |

1. How many irrigation cycles are typically involved in conventional and organic cultivation?
2. What is the average water and electricity consumption for irrigation, and how is water sourced?

# D. OTHER AGRICULTURAL PRACTICES

1. Operations in the field

| ***Type of field operations*** | ***Total working hours*** | ***Number of employees*** | ***Time Period***  ***eg. From October to November*** | ***Quantity of Fuel used in Agricultural Machinery*** |
| --- | --- | --- | --- | --- |
| Tillage-weeding |  |  |  |  |
| Hoeing |  |  |  |  |
| Planting of nursery |  |  |  |  |
| Mechanical pruning |  |  |  |  |
| Chemical fertilizer  application |  |  |  |  |
| Chemical fertilizer  application |  |  |  |  |
| Pesticide Spraying |  |  |  |  |
| Irrigation |  |  |  |  |
| Harvesting |  |  |  |  |

1. Operations outside the Field

| ***Type of operations*** | ***Total operation hours of machinery per year*** | ***Type of machinery*** | ***Cost per kg of marketable product*** | ***Other*** |
| --- | --- | --- | --- | --- |
|  |  |  |  |  |

Total cost until the final product (in INR)
